# Effect of Annealing Temperature on ECD Grown Hexagonal-Plane Zinc Oxide

**DOI:** 10.3390/ma11081360

**Published:** 2018-08-06

**Authors:** Sukrit Sucharitakul, Rangsan Panyathip, Supab Choopun

**Affiliations:** Department of Physics and Materials Science, Faculty of Science, Chiang Mai University, 239 Huay Keaw Road, Muang Chiang Mai 50200, Thailand; sukrit.sucharitakul@gmail.com (S.S.); rangsanpanyatip@gmail.com (R.P.)

**Keywords:** ZnO, hexagonal, electrochemical deposition

## Abstract

Zinc oxide (ZnO) offers a great potential in several applications from sensors to Photovoltaic cells thanks to the material’s dependency, to its optical and electrical properties and crystalline structure architypes. Typically, ZnO powder tends to be grown in the form of a wurtzite structure allowing versatility in the phase of material growths; albeit, whereas in this work we introduce an alternative in scalable yet relatively simple 2D hexagonal planed ZnO nanoflakes via the electrochemical deposition of commercially purchased Zn(NO_3_)_2_ and KCl salts in an electrochemical process. The resulting grown materials were analyzed and characterized via a series of techniques prior to thermal annealing to increase the grain size and improve the crystal quality. Through observation via scanning electron microscope (SEM) images, we have analyzed the statistics of the grown flakes’ hexagonal plane’s size showing a non-monotonal strong dependency of the average flake’s hexagonal flakes’ on the annealing temperature, whereas at 300 °C annealing temperature, average flake size was found to be in the order of 300 μm^2^. The flakes were further analyzed via transmission electron microscopy (TEM) to confirm its hexagonal planes and spectroscopy techniques, such as Raman Spectroscopy and photo luminescence were applied to analyze and confirm the ZnO crystal signatures. The grown materials also underwent further characterization to gain insights on the material, electrical, and optical properties and, hence, verify the quality of the material for Photovoltaic cells’ electron collection layer application. The role of KCl in aiding the growth of the less preferable (0001) ZnO is also investigated via various prospects discussed in our work. Our method offers a relatively simple and mass-producible method for synthesizing a high quality 2D form of ZnO that is, otherwise, technically difficult to grow or control.

## 1. Introduction

ZnO has been widely studied as a platform for a broad spectrum of applications. Thanks to the material’s widely variable electrical and optical properties, based on its nanostructure types or effective planes align, the material can be a strong candidate for biosensing [1], ultraviolet lasing [2], varistor [3], catalysts [4], optoelectronic devices [5] and, more importantly, photovoltaic cells [6,7,8,9,10,11,12,13]. ZnO has attracted attention from the research community. For the past decade, there have been many advancements on the material’s growth and synthesis techniques, mostly focused on ZnO’s nano particle forms; namely, ZnO nanowire, which is the 1-dimensional case. Techniques have been developed to improve the yield and control over the grown material’s morphology, ranging from chemical-vapor deposition [14,15,16], pulse laser deposition [17,18], hydrothermal growth [19,20], vapor–liquid–solid reaction [21,22,23], microwave heating [24], precipitation method [25], sonochemical method [26,27], and electrochemical deposition [28,29,30,31].

As mentioned, despite tremendous advancements of ZnO growth in bulk, nanodots, and nanowire forms through various techniques, the 2-dimensional formats of ZnO have remained relatively unexplored. Not until the recent discovery by Novoselov et al. of 2-dimensional exfoliable materials [32], have the community’s interests started shifting towards the regime of 2-dimensional materials. This has inspired the community to focus its attentions to further investigate ZnO in the 2-dimensional form, both experimentally [30,33,34,35,36,37,38] and theoretically [39,40].

Interestingly, it has been shown that ZnO in the 2-dimensional form of (0001) or hexagonal plane (referred to as h-ZnO for the rest of this work) can be more effective in certain aspects than all its other forms (e.g., quantum dots, nanowires, and bulk) thanks to the plane being more polarized, which allows the plane to be significantly more sensitive to polar molecules and light interactions, and thanks to the higher ratio of the materials in such a plane. Furthermore, the theoretical results suggested that charge carriers have a higher mobility and lower combination rate in such planes, making h-ZnO not only an interesting platform for scientific studies but also a very strong candidate for Photovoltaic (PV) applications.

In growing h-ZnO, there have been reports regarding the success of the growths via various methods. The list includes pulse laser deposition (PLD) [35], commercial Simonkolleite annealing [36], hydrothermal method [37], and electrochemical depositions (ECD) [9,28,30,31,33,34,41]. However, in the report listed so far, there have been no methods that can grow atomically thin ZnO with a high aspect ratio on a hexagonal plane with scalable growth techniques. In this work, h-ZnO crystals are grown via ECD, followed up by thermal annealing to maximize the growth and improve the aspect ratio prior to further optoelectrical investigations. The method proposed in this work combines the strength of a relatively simple electrochemical deposition, which allows mass production of flake nucleation in highly controlled direction, with the quality and grain size offered by the followed-up annealing, which will be discussed further in the later part of this paper.

## 2. Sample Preparation and Methodology

**Figure 1:**
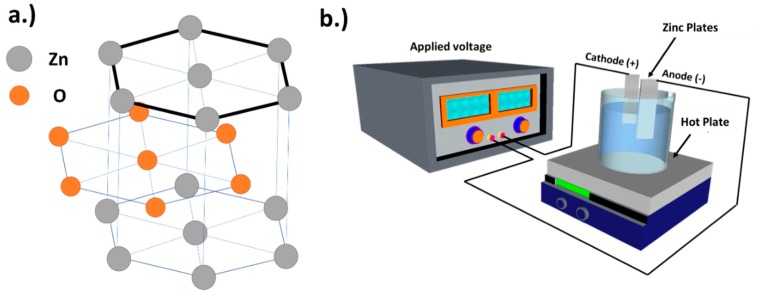
Illustration of (**a**) crystal structure of ZnO highlighting the (0001) plane; and (**b**) electrochemical setup used in this work.

In this work, the authors propose the procedures for growing h-ZnO via electrochemical deposition prior to annealing at a higher temperature for the promotion of growth in (0001) plain as shown if Figure 1a. The procedure starts with pre-cleaned cut Zn plates sonicated in Acetone for 10 min prior to rinsing in Ethanol, DI water then blown dry prior to being used as electrodes.

### 2.1. Materials

In mixing electrolyte solution for the growth, commercially purchased Sigma-Aldrich (St. Louis, MO, USA) 99.99% grade Zn(NO_3_)_2_ and KCl powder were mixed with DI water in a standard 100 mL beaker seated on hot plate.

### 2.2. Preparation of Hexagonal-Plane Zinc Oxide Via Electrochemical Deposition

The growth conditions are set to be similar to work conducted by Pradhan et al.; initially, the bath’s electrolyte is first mixed with 0.04 M of Zn(NO_3_)_2_ and 0.1 M of KCl in deionized water (DI) at room temperature, then the bath is preheated until the temperature is at an equilibrium at 45 °C as read by the DS18b20 waterproof thermal sensor. Agilent E3633A power supply is then set in direct current (DC) voltage bias mode with 1.1 V potential difference between the Cathode and the Anode as demonstrated in the figure as the growth commences. Commercial grade Zn plates were cut into 1 cm × 5 cm pieces via metal cutter and used as electrodes for the growth process. Grown crystals are then extracted from the solution for further investigation and annealed at various temperatures on a hot plate in an ambient environment with calibrated equilibrium temperature. The process of material preparation was systematically studied through Cyclic voltammetry over ranges of electrolyte concentration of 0.1 M–0.5 M before 0.1 M was picked as the growth condition (the reason is discussed later in the article) while the growth period is a controlled 30 min, followed by annealing at different temperatures ranging from 100 to 300 °C for the final product. The annealing was done at ambient gas pressure and composition to provide the oxygen for material’s composition as discussed later in the article. The appropriate analysis and characterizations are separated into different stages of growth to gain insights on the grown material’s properties in each stage in order to demonstrate insights on the growth process.

### 2.3. Characterization and Analysis of the Obtained Materials

In this work, in order to quantify and qualify the materials, electron microscopic and spectroscopic techniques were used. For scanning electron microscope (SEM) and electron diffraction spectroscopy (EDS) techniques, the results were collected using a FE-Scanning Electron Microscope: JSM 6335 F while all information obtained using tunneling electron microscopy, the JEOL JEM-2010 TEM model (MA, USA) was utilized on synthesized samples collected on standard Cu grids.

**Figure 2:**
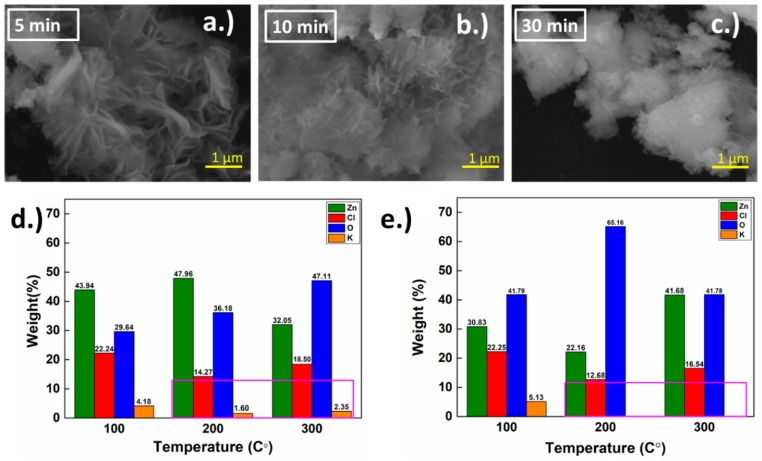
Scanning electron microscope (SEM) images of the extracted crystal grown for (**a**) 5; (**b**) 10 and (**c**) 30 min and electron diffraction spectroscopy (EDS) results of ZnO crystals grown (with K element as weighing factor in pink rectangle) for (**d**) 5 and (**e**) 30 min).

Immediately after the growth was finished, the h-ZnO crystals were extracted and investigated via SEM and EDS to gain insight on the immediate composition after the growth and prior to annealing. Results are as demonstrated in Figure 2d,e.

As illustrated in Figure 2a–c, growth starts initially with K^+^ deposition in competition with Zn^2+^ onto the Cathode. K^+^ deposits better than Zn^2+^ thanks to its higher mobility giving the structure rather complicated shape initially as shown in 5 min. The crystals start growing more uniformly and showing more rigid domains of vertical h-ZnO growth as time goes on, forming clusters on the substrates. This is incompatible with previous work [33], where grown flakes are in the order of a few microns. Our initial growths demonstrate the role of Cl^−^ as a capping ion, distinguishing and allowing the (0001) plane to grow more energetically and favorably, in accordance with arguments and demonstrations proposed in previous works [33,42]. From Figure 2d,e, the EDS results further demonstrate the role of K^+^ and Cl^−^ in the growth process. Firstly, K^+^ initially deposited on Cathode with a higher relative ratio compared to Zn^2+^ thanks to the ions’ higher mobility in the solution. From our observation, this is generally reduced as the growth time increases (for in depth discussions regarding this topic, please refer to our Supplementary Material). While Cl^−^ ions facilitate the mobility of Zn^2+^ and the remaining Cl^−^ ions in the solution are expected to deposit on the final product after the solvent part of the supernatant is evaporated. It is noteworthy that although Cl^−^ ions are functioning as intended as a facilitator of Zn^2+^ deposition and capping of the (0001) plane on ZnO growths, they are also considered unwanted defects when the h-ZnO flakes are used for PV application, as it reduces mobility and increases the recombination rate of carriers. Thus, we propose to anneal the products after the growth, which will be discussed later in the article.

## 3. Growth Mechanism Discussions and Further Annealing

In order to obtain h-ZnO structures with high yield, Cl^−^ ion had been attributed as the “capping ions”, which becomes strongly attracted to the (0001) plane whose polarization is stronger than the other 2D planes of ZnO, while being less energetically favorable to growths [33,42]. Although the mechanism for such growths has undergone extensive debate, so far no conclusive evidence has been unearthed. For further qualitative understanding on the role of KCl in h-ZnO growth, the authors have performed cyclic voltammetry (CV) utilizing the ER461 EChem Startup System on the KCl solution in the setup used in this work to determine the role of the K^+^ and Cl^−^ ions in the growth contribution in the growth setup. It is noteworthy that in the results the anodic oxidation and cathodic reduction peaks were barely observed during the scan range, all of which falls within the 1.1 Volt, which is the growth voltage [43]. 

### 3.1. The Primary ZnO Growth

The nucleation process of the electrochemical method takes place prior to more complicated secondary processes. The rate of this growth process depends on the reaction rate (oxidation and reduction rate) on the surface electrode (Zn) in the electrochemical deposited method [44]. The growth mechanism of ZnO in the primary process has the reaction process with Equation (1), which can be separated into the reactions as the cathode process, such as in the equation listed below (2–8) while the anode was extracting, Equation (9) defines the aqua ion in reaction. The overall reaction for nucleation was dependent on the deposition process to the cathode surface. Hence, considering the primary growth reaction of ZnO via ECD with Zn(NO_3_)_2_ ($\mathrm{Zn}{\left({\mathrm{NO}}_{3}\right)}_{2}6H_{2}O$) and KCl as reagents, they can be broken down in the chemical reactions listed below [45,46,47,48]:

(1) $$\mathrm{Zn}{\left({\mathrm{NO}}_{3}\right)}_{2}\;+\;2\;\mathrm{KCl}\;⇋{\;\mathrm{ZnCl}}_{2}\;+\;2{\;\mathrm{KNO}}_{3}$$

Cathode:

(2) $$\mathrm{Zn}{\left({\mathrm{NO}}_{3}\right)}_{2}\to {\mathrm{Zn}}^{2+}+2{\mathrm{NO}}_{3}^{-}$$

(3) $$\mathrm{KCl}\to K^{+}+{\mathrm{Cl}}^{-}$$

(4) $${\mathrm{NO}}_{3}^{-}+H_{2}O+2e^{-}\to {\mathrm{NO}}_{2}^{-}+2{\mathrm{OH}}^{-}$$

(5) $${\mathrm{Zn}}^{2+}+2{\mathrm{OH}}^{-}\to \;\mathrm{Zn}{\left(\mathrm{OH}\right)}_{2}$$

(6) $$\mathrm{Zn}{\left(\mathrm{OH}\right)}_{2}\to \mathrm{ZnO}+H_{2}O$$

(7) $${\mathrm{Zn}}^{2+}+2{\mathrm{Cl}}^{-}\to \;\mathrm{Zn}{\left(\mathrm{Cl}\right)}_{2}$$

(8) $$K^{+}+{\mathrm{NO}}_{3}^{-}\to {\mathrm{KNO}}_{3}$$

Anode:

(9) $$H_{2}O\to \frac{1}{2}O_{2}+2H^{+}+2e^{-}$$

Primary growth of ZnO is the heterogeneous reaction between the bulk solution (electrolyte) and electrode surface cations. The anions in the electrolytes are separated by electrical activation forming reactions on the electrode as shown in the equations. This nucleation mechanism is strongly influenced by electron transfers near the electrode surface and electrolyte. Via the observation of the ratio $I_{P\left(\mathrm{re}\right)}/I_{P\left(\mathrm{ox}\right)}$, further insights in the nucleation mechanism can be obtained [49], where the redox reaction in an electrochemical process can be determined with the maximum current ($I_{P}$) quantified from the CV plot and the Randles–Sevcik Equation (10) [49]:

(10) $$I_{p}=0.446\mathrm{nFAC}{\left(\mathrm{nFDv}/\mathrm{RT}\right)}^{1/2}$$

where I_p_ is current maximum in amps, n is number of electrons transferred in the redox event, A is electrode area in cm^2^, F is Faraday constant in C mol^−1^, D is diffusion coefficient in cm^2^/s, C is concentration in mol/cm^3^, v υ is scan rate in V/s and R is gas constant in JK^−1^ mol^−1^ and Tis temperature in K. Further discussion about energetic and kinetic information of the reaction can be found in our Supplementary Material Section S1 (c.f. Figure S1). 

**Figure 3:**
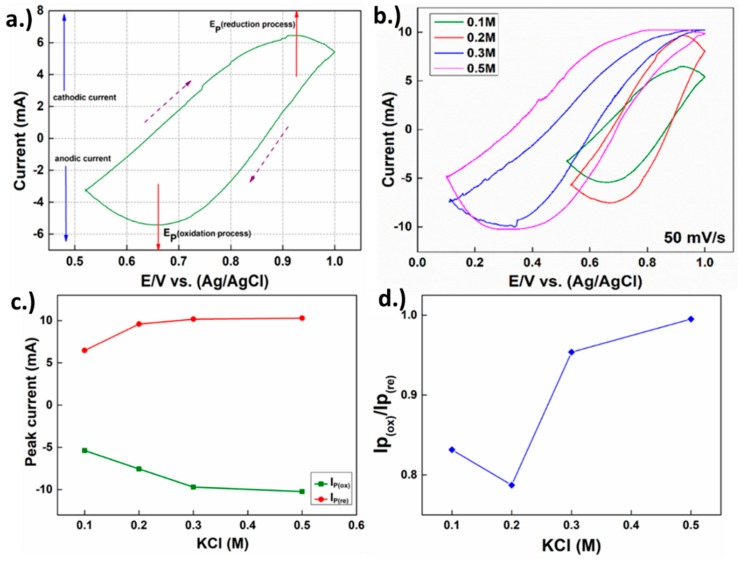
Cyclic voltammetry (CV) measurement of (**a**) ZnO with 0.1 M of KCl; and (**b**) ZnO growth dependence of 0.1 M–0.5 M for KCl concentration. The effect of KCl concentration in cyclic voltammetry to growth ZnO; (**c**) peak oxidation and reduction in each KCl concentration; (**d**) ratio of redox reaction vs KCl concentration ranging from 0.1 M to 0.5 M.

As pointed out in the main article the cyclic voltammetric data represents the reversible process and fast electron transfer behavior [49] through the cyclic voltammogram in hexagonal ZnO shown in Figure 3a,b of the main article. The hysteresis offers evidence that the redox process is strongly dependent on the rate chosen for the measurement. The area of voltammogram was separated as the anodic current oxidation process and cathodic current reduction process, both of which are components used to discuss the synthesized hexagonal ZnO, therefore, the electrochemical mechanism in Figure 3a started at 0.64 V, as indicated by the slope of the growth up to 1.0 V, reaching maximum cathodic current $I_{P\left(\mathrm{re}\right)}$ for 6.47 mA at 0.93 V, then the reversible process began as the voltage was ramped to 1.0 V and reached the oxidation zone at 0.64 V, which observed the maximum anodic current $\left(I_{P\left(\mathrm{ox}\right)}\right)$ of 5.41 mA at 0.66 V. As previously discussed in an earlier study [49], the rate of nucleation on the electrode surface can be influenced completely differently based on how far the nucleation actually takes place from the surface, which can be separated into 3 zones: the bulk solution zone (electrolyte zone), diffusion layer zone (~1 nm to 500 µm form electrode surface), and compact layer zone, which is directly on the surface up to approximately 0.1 nm thick. The physics that separates the compact layer (a.k.a. Inner Helmholtz) from the diffusion layer (a.k.a. Gouy–Chapman) is the saturation of the diffusion range where the growth mechanics in the first are determined by solvent’s dipole moment as that of the latter is strongly affected by the electron transfer and diffusion rate [49]. 

This model can be used to explain the nucleation-growth mechanism in the ZnO single-layer crystal. From the CV results in Figure 3a in the main article and as discussed in the supplemental material section SI, the role of the KCl concentration on the electron transfer rate can be inferred via ($I_{P\left(\mathrm{re}\right)},I_{P\left(\mathrm{ox}\right)}$) since electron transfer is strongly influenced by the KCl concentration, allowing us to somewhat control the electron transfer rate by the variation of (KCl) while observing the ratio of $I_{P\left(\mathrm{re}\right)}/I_{P\left(\mathrm{ox}\right)}$. Based on our results in the main article demonstrated in Figure 3b, at (KCl) ranging from 0.3 M to 0.5 M demonstrates high electron transfer phenomena. The reason of this process was the induced ion diffusion to electrode surface with a relatively high diffusion rate as a result of the higher concentration of the ions, resulting in a wider diffusion range compared to the 0.1 M and 0.2 M as can be implied from the peak current and the ratio of $I_{P\left(\mathrm{re}\right)}/I_{P\left(\mathrm{ox}\right)}$ demonstrated in Figure 3. However, such an effect is not favorable for continuous growth in the secondary process as the uniformity can be reduced with a higher growth rate.

To demonstrate the previously explained growth mechanism, SEM images of ZnO crystal grown under our standard condition with (KCl) as 0.1 M were taken at different growth times and demonstrated in Figure S2 (c.f. Supplementary Material). It can be inferred from Figure S2a that the secondary growths already took place after 30 s into the growth, the vertical crystal seeded from the compact layer grew correspondingly with time, as can be seen in Figure S2b. After 300 s of growth, the flakes had a dense uniformity to a bulk layers as Figure S2c [41] and Figure S2d where the saturation of ZnO crystal growth in forms of bulk flakes [50], which can improve the crystal sizes with annealing temperature in the next process.

As demonstrated in Figure 3a, the maximum current ratio of cathodic and anodic $\left(I_{P\left(\mathrm{re}\right)}/{\;I}_{P\left(\mathrm{ox}\right)}\sim 1\right)$ in the electrochemical process can be extracted and utilized to reveal the fast electron transfer mechanism during the reversible reaction of reduction and oxidation redox respectively. Moreover, the mechanism can be used to explain the role of KCI in the electrochemical process by testing the reaction with a KCl concentration of 0.1 M–0.5 M during the synthesis of h-ZnO. It is noteworthy that without the usage of KCl into the solution, reactions (c.f. Supplementary Material for further details) leading to h-ZnO flakes cannot take place, this is due to the lack of catalyst to support the ion for redox reaction with an electrode. On the other hand, after KCl is used for 0.1 M in the electrochemical process ZnO can be produced and then by increasing the KCl concentration from 0.2 M to 0.5 M revealed CV, the results of which are shown Figure 3b, in which the comparison of CV and ZnO in each KCl concentration is demonstrated. It can be inferred that when the KCl concentration is more than 0.1 M (e.g., 0.2 M–0.5 M), the CV plot’s area increases with the concentration, as can be seen from our results. This can be attributed to the relatively higher redox reaction rate enabled by the higher concentration of the K^+^ and Cl^−^ ions compared to the 0.1 M case, while the latter allows a higher mass-producibility and uniformness of the h-ZnO flakes [51,52]. As previously discussed in the EDS section, there was evidence to suggest that K^+^ and Zn^2+^ compete to grow as crystals, which the EDS result confirmed: K^+^ deposited faster than Zn^2^^+^ [52], especially in the early stage. Additionally, excessive amounts of KCl could promote other growth species, such as KNO_3_ and ZnCl_2_ while 0.1 M is proven to be sufficient for the purpose of capping the (0001) plane [52]. Thus, 0.1 M was chosen as the standard growth condition in this work [45,53]. Through extraction of the reduction current ($I_{P\left(\mathrm{re}\right)}$) and oxidation current ($I_{P\left(\mathrm{ox}\right)}$) from Figure 3b into Figure 3c, there is a clear enhancement trend shown with increasing KCl concentration, while presented in Figure 3d is the ratio of $I_{P\left(\mathrm{ox}\right)}/I_{P\left(\mathrm{re}\right)}$, which remains relatively constant as a function of [KCl]. It is also noteworthy to point out that during the cyclic voltammetry, two channels are compared in Figure 3b; the change of effective hysteresis differential conductance is clearly observable. This may imply that the usage of 0.1 M, as suggested by previous work [33], has a strong hysteresis, allowing the reverse of the differential conductance at a 100 mV/s scan rate while the concentration increases and the hysteresis is reduced. This demonstrates that given 0.1 M, the KCl concentration used in the actual growth would have a strong time dependence, especially during the beginning of the growth, allowing non-equilibrium deposition of K and Cl to take place explaining the inhomogeneity of our growths at lower growth time, as shown in Figure 2a. In addition, h-ZnO CV showed superior electrochemical performance and stability at high current density with low voltage, thus it is suitable for solar cell applications [45].

### 3.2. The Secondary Growth 

This process takes place after the nucleation of the compact layer is finished. The secondary growth can be attributed to the vertical growths that are observed in the main article. This process is similar to the coalescence behavior of two droplets of water when they are connected and then form one bigger droplet instead of staying separately, hence the name of the process. Through this process, the growth mechanism combines the nucleation grains of ZnO from primary growth to the forming of large scale of particles that form flake sizes through the coalescence process [54,55,56,57]. 

To further shed some light on the role of the coalescence process and the effectiveness of 0.1 M of KCl concentration in the growth of h-ZnO flakes, Gibs free energy needs to be considered. Through Equation (11), one can calculate the Gibs free energy change of the nucleation process:

(11) $$\Delta G_{N}^{*}=4{\pi r}^{*}{}^{2}\gamma_{f}+\frac{4}{3}{\pi r}^{*}{}^{3}\Delta G_{v}$$

where, $\gamma_{f}$ is surface energy of nucleation atom, r* is effective critical radian of atom nucleation (unitless) and $\Delta G_{v}$ is free energy of the bulk crystal (Gibbs free energy of volume growth).

With the known change of Gibbs free energy change, the probability of coalescence process to occur in secondary growth (P_N_) [58] with each annealing process where in the case that the change in Gibbs free energy of the process is given by $\Delta G_{N}^{*}$ can simply be calculated via Equation (12) as follows

(12) $$P_{N}=e^{\left(-\Delta G_{N}^{*}/k_{B}T\right)}$$

where, k_B_ is the Boltzmann constant and T is the temperature of the growth in the process.

Thus, from our experimentally obtained data, one can determine the value of P_N_ of ZnO nucleation in 0.1 M (KCl) case at various annealing temperatures from previously calculated $\Delta G^{{}^{\circ}}$ as shown in Figure S1 in the Supplementary Material.

Thus, by using Equation (12) through $\Delta G^{{}^{\circ}}$ information previously obtained in Figure S1a, the coalescence probability can be calculated via Equation (12). As the annealing temperature increases, one can infer that the recombination rate is also increased; e.g., higher final $\Delta G^{{}^{\circ}}$. Moreover, the grains were influenced by high $\Delta G^{{}^{\circ}}$ after the ZnO flake was grown up with the annealing process [59] to 31.97 ${\mu m}^{2}$, 52.01 ${\mu m}^{2}$ and 437.79 ${\mu m}^{2}$, respectively, as shown in Figure S1b and discussed in section SI of our Supplementary Material.

Within the range of our trials, since the 0.1 M concentration has the lowest reaction rate in the primary growth process and has high $\Delta G^{{}^{\circ}}$ in the secondary growth process, we were thus led into choosing 0.1 M of KCl as our default growth choice in order to enhance the hexagon ZnO flake sizes with the annealing method for improving the degree of ZnO crystallinity [60]. Through this agglomeration process in combination with secondary nucleation on top of the ZnO seeding molecules formed in the primary growth, ZnO crystals, as shown as the final product in Figure 2c, can be obtained.

### 3.3. Annealing of Grown h-ZnO Nanoflakes

The annealing of ZnO from Simonkolleite in ambient in order to obtain large h-ZnO nanoplates was realized and brought to light by Zhang et al. [61]. Thanks to the precursor, Simonkolleite, which is also a hexagonal crystalline precursor, the process can provide high quality h-ZnO, however, the shape and the crystal aspect ratio of the resulting crystals are less controlled, albeit with porous features left behind by the chloride hydroxide monohydrate chains diffusing out of the material, leaving behind porous features at a higher temperature. In this work, such a method is applied to the grown crystals, allowing a higher aspect ratio of (0001) plane while keeping the crystallinity through the process while starting from h-ZnO as a precursor, allowing the relocations and diffusion of Zn and O atoms to become more favorable and controlled. Additionally, we believe that the coalescence process played a strong role in dictating the grown crystal size (c.f. Section SI of Supplementary Material).

**Figure 4:**
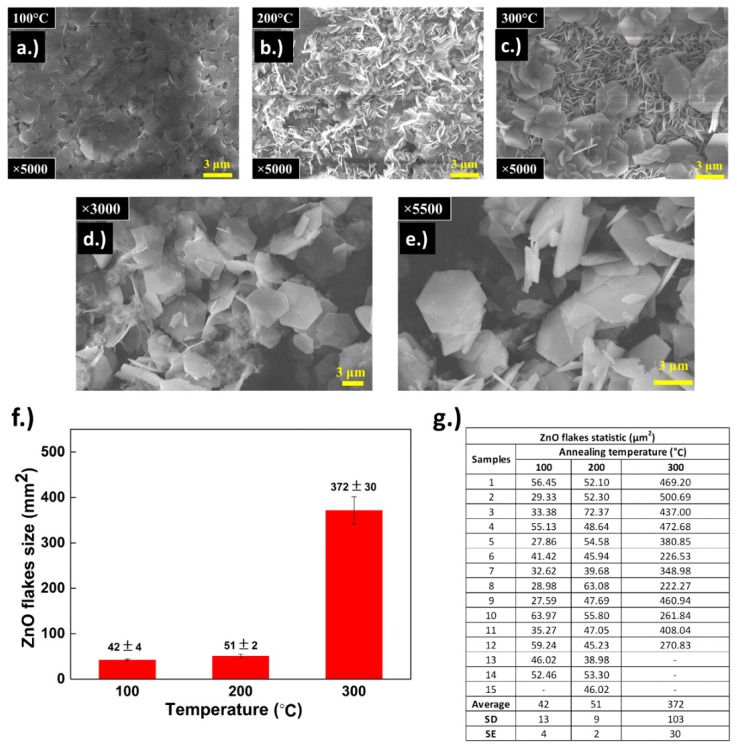
SEM images of the grown ZnO crystals grown for 30 min prior to annealing at (**a**) 100; (**b**) 200; and (**c**) 300 °C for 1 h; and (**d**,**e**) more SEM images at different magnifications and locations for flakes annealed for 300 °C for 1 h for comparison; (**f**) statistical summary of (0001) plane flake size demonstrating statistics of flakes annealed at different temperature; and (**g**) flake’s statistics at different annealing temperature (rounded down to decimal points.

As illustrated in Figure 4a–c the annealing temperature does play an important role in increasing the size of (0001) plane. It is noteworthy that accidental doping by both Cl^−^ and K^+^ can be fixed via annealing. Since the annealing is conducted in an ambient environment, K^+^ is treated and removed by humidity, thus, the reduction of K^+^ ion while Cl^−^ effectively capped the ZnO on the (0001) plane, making the ions difficult to remove. As demonstrated in Figure 4c, annealing at higher temperature gives higher thermal energy for the already deposited Zn^2+^ and O^2−^ to decompose and recompose into new seeded h-ZnO, allowing for the composition of larger final annealed flakes. As demonstrated in the EDS results, the K^+^ component is either decomposed or becomes less detectable due to the yield and size of the ZnO flakes. It is also noteworthy to point out that the h-ZnO prepared this way has less Cl contamination than that prepared by the annealing of commercial Simonkolleite, allowing for grown crystals superior in terms of electron trapping for PV devices, especially in the case of nano-scale flakes [62], and efficiency as a gas sensor [63]. Also, by annealing only up to 300 C, the grown flakes have less tendency to become porous [64] and, thus, have a higher carrier mobility in PV applications, albeit such properties could also be beneficial for a gas sensing application. Furthermore, this proposed method allows significantly larger grown flakes, as demonstrated in Figure 4f, despite them being less favorable thermodynamically according to our Gibbs free energy analysis (c.f. Section SI of Supplementary Materials).

### 3.4. Post-Annealing Characterization

**Figure 5:**
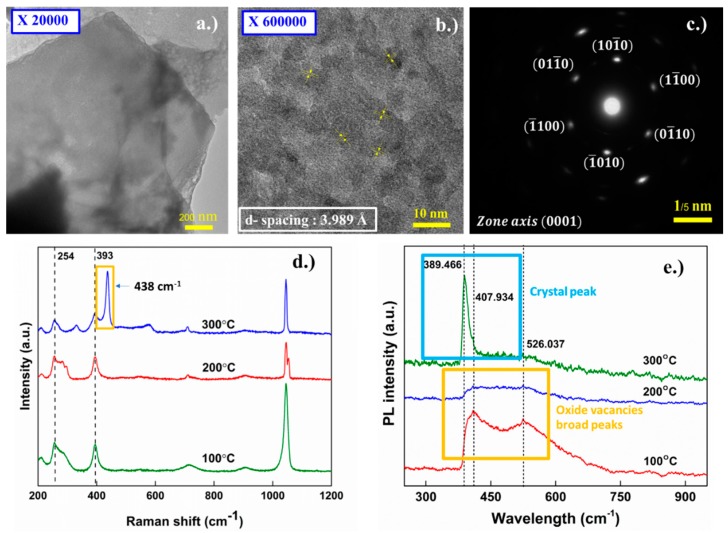
Transmission electron microscopy (TEM) image at (**a**) lower; and (**b**) higher resolution of ZnO flakes grown for 30 min prior to annealing at 300 °C for 1 h in ambient environment; (**c**) Electron diffraction and zone analysis for the crystals; and (**d**) Raman spectrum and; (**e**) photoluminescence (PL) results of flakes grown for 30 min then annealed at different temperatures.

To further confirm the quality of the annealed samples, a series of tests were conducted to verify the integrity of the final products from our proposed process, TEM was first used to characterize the crystallinity of the sample. Figure 5a–c illustrated TEM results on the annealed crystals, which were grown for 30 min prior to annealing at 300 °C for 1 h in an ambient environment. The d-spacing of the crystals was determined to be 3.989 Angstroms and the zone analysis patterns demonstrates the high crystallinity in the grown samples, which is in good agreement with previous work [65]. As illustrated in Figure 4e,f, from the Raman results on the annealed crystals, a prominent 438 cm^−1^ E_2_ peak corresponding to (0001) plane was detected at 300 °C annealing. This peak was less observed in lower annealing temperature, likely due to a different orientation of the (0001) plane whilst the planes are grown vertically. A small shift of 393 cm^−1^ and 254 cm^−1^ peaks were observed, this is likely due to the change in size constraints as the flakes grow larger with the annealing temperature, relaxing the average mode of the phonons in a similar case to the quantum dots case previously studied by Cheng et al. [38]. As shown in Figure 4f, the photoluminescence demonstrates the superiority of the crystals annealed at 300 °C, as a crystal peak at 389 nm wavelength is clearly dominant. We attribute the broadening of the PL peaks on our samples to oxygen vacancies at lower annealing temperature. Again, this confirms our assumption of reduction of defects of accidental doping by the electrochemical deposition process with higher temperature annealing in ambient and the results are also in good agreement with a discussion provided by Zhang et al., 2017 [61] regarding the annealing temperature dependence trend and a device’s performance. In addition, our results also suggested that in work done by Zhang, the poor performance of flakes annealed at lower temperature may have been due to the oxide vacancies, as can be observed in our optical results. Regardless, such issues can be avoided through our proposed method of using ECD grown h-ZnO nanoplates as a precursor instead of Simonkolleite.

## 4. Conclusions and Outlook

In this work, a relatively simple and scalable process for h-ZnO crystals growth for PV application via ECD in combination with annealing is proposed. Through observations via SEM images, we have analyzed the statistics of the grown flakes’ hexagonal plane’s size. Our results showed a non-monotonal strong dependency of the average flake’s hexagonal flakes’ size up to the average of 300 µm^2^ at 300 °C annealing temperature as shown in Figure 4f, which is a large improvement on the (0001) preferred plane growth via a similar method. The roles of Cl^−^ and K^+^ ions were investigated via CV measurements. Through optoelectrical analysis via Raman spectroscopy, SEM and TEM, the high-quality h-ZnO crystals grown were confirmed and the role of affiliating ions in electrochemical growth mechanisms were further understood. Furthermore, through our I-V measurement, we have demonstrated the potential of h-ZnO flakes as a platform for PV based device applications via a low heat preparation process (c.f. our Supplementary Material Section SII for electrical test results). This process not only provided high quality h-ZnO crystals but also holds promises for a mass-producibility transition for industries thanks to the method’s relative simplicity and scalability. And finally, through this work, we have provided further insight into the growth mechanisms of h-ZnO in the ECD process and the role of assistive ions such as K^+^ and Cl^−^ in such mechanisms detailed in our 2-step growth model.

## Supplementary Materials

The following are available online at http://www.mdpi.com/1996-1944/11/8/1360/s1. Figure S1. Plot of calculated (a) the reaction rate in equilibrium K_C_ and the variable of Gibbs free energy level ($\Delta G^{{}^{\circ}}$) versus KCl concentration and (b) the h-ZnO’s nanoplatelet sizes’ plotted with $\Delta G^{{}^{\circ}}$. Figure S2. SEM image of grown h-ZnO flakes on the electrode at (a) 30 s, (b) 120 s, (c) 300 s and (d) 1800 s into the growth. Figure S3. Illustration of (a) the device fabricated for deposited ZnO flakes, (b) I-V curve of the ZnO nanoflakes device with [KCl] = 0.5 M and (c) the calculated conductance of ZnO device vs [KCl] used in the synthesis.
