# Supplementary material for: Effect of Annealing Temperature on ECD Grown Hexagonal-Plane Zinc Oxide
Source: Materials (Basel). 2018 Aug 6;11(8):1360. doi: 10.3390/ma11081360 (PMC6120048; doi:10.3390/ma11081360)
Supplement: Supplementary file 1 [file materials-11-01360-s001.pdf]

materials

MDPI

Article's Supplementary Materials

Supplementary Materials: The Effect of Annealing Temperature on ECD Grown Hexagonal-Plane Zinc Oxide

Sukrit Sucharitakul [1], Rangsan Panyathip[1] and Supab Choopun [1,*]

[1] 1 Department of Physics and Materials, Faculty of Science, Chiang Mai University, 239 Huay Keaw Road, Muang Chiang Mai, 50200, Thailand

* Correspondence: supab99@gmail.com; Tel.: +66-819-512-669

Academic Editor: < >

Received: < >; Accepted: < >; Published: < >

SI: The effect of KCl Concentration on Growth Parameters

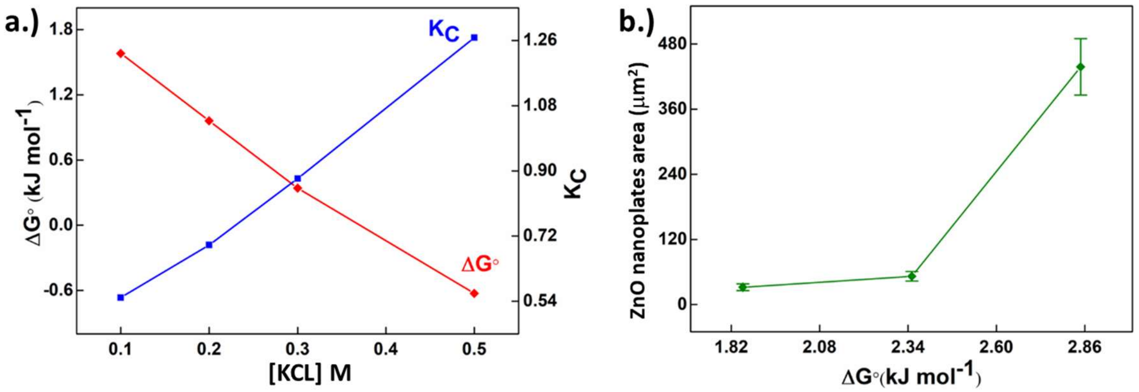

Figure. S1 Plot of calculated a.) the reaction rate in equilibrium K_C and the variable of Gibbs free energy level (ΔG°) versus KCl concentration and b.) the h-ZnO's nanoplatelet sizes' plotted with ΔG°

This section of supplementary materials discusses the role of KCl and annealing temperature in h-ZnO growths . As shown in Figure S1.a.), the relativity of thermodynamic equilibrium constant (K_C) was calculated via equation (2) and plotted versus the concentration of KCl in our experimental range (0.1M-0.5M) to gain further insights on its effects on the growth of h-ZnO. In this investigation, the concentration of KCl is used as primary variable to figure out other parameters. K_C is then calculated to determine the reaction rate in equilibrium based on the final concentration . As for the results, ZnO grown from 0.1M, 0.2M and 0.3M had reaction rate at 0.55, 0.6955 and 0.879 respectively. Since all K_c were determined to be less than unity, it means the reaction remains sub-equilibrium while in 0.5M case the Kc was determined to be 1.2678, whilst, this behavior is consistent with CV results from earlier .

$$Zn(NO_3)_2 + 2KCl \leftrightharpoons ZnCl_2 + 2KNO_3 \quad \text{(SI1)}$$

$$K_C = \frac{[ZnCl_2][KNO_3]^2}{[Zn(NO_3)_2][KCl]^2} \quad \text{(SI2)}$$

Typically, in growing homogeneous and large flakes, it's more preferable to stay well under equilibrium kinetically so that the thermodynamic can have sufficient time to kick in to the reaction,

which will be discussed in the next section of the supplementary materials, and such is why 0.1M of KCl was determined to be optimal for growing (0001) ZnO crystal in our case.

When discussing thermodynamics of the h-ZnO crystal growths, Gibbs free energy of the reaction in equilibrium (ΔG°) can be used to determine the favorability of nucleation in h-ZnO with an electrochemical process which it defined the lowest energy to excited for reaction .This Gibbs free energy term was implicated with the reaction rate constant shown in equation (3):

$$\Delta G^{\circ} = -RTlnK_C \quad \text{(SI3)}$$

Where R is gas constant $Jmol^{-1}$and temperature (T) The considered ΔG° in equilibrium system contributed to mechanism or nucleation of ZnO in solution as equation (4) which this relation was included ΔG° of nucleation surface ($\Delta G_{surface}$) and ΔG° of nucleation volume.

In order to determine the spontaneity of the reaction, ΔG° was calculated using equation (SI3) through the calculated K_c values of the corresponding case as shown in Figure SI1. Based on our results at 0.5M of KCl ΔG° is -0.627 $kJmol^{-1}$ making the reaction spontaneous while for 0.1M,0.2M and 0.3M of KCl composition had ΔG° for 1.58 kJ mol^{-1} 0.96 kJ mol^{-1} and 0.34 kJ mol^{-1} respectively (as shown in Figure .S1). Moreover, since in 0.1M -0.3M cases, $\Delta G^{\circ} > 0$, this reaction required another parameter to excitation process with either applied voltage or annealing temperature. Furthermore, based on our growths and annealing of h-ZnO flakes at different conditions shown in Figure 4.f.), presented in Figure SI2 is the calculated ΔG° for each flake sizes via equation (3).

Generally, nucleation atom was assumed to sphere shape, therefore, ΔG° in nucleation atom in ZnO as defined in equation (5)

$$\Delta G^{\circ} = \Delta G_{surface} + \Delta G_{volume} \quad \text{(SI4)}$$

$$\Delta G^{\circ} = 4\pi(r^*)^2\gamma + \frac{4}{3}\pi(r^*)^3\Delta G_v \quad \text{(SI5)}$$

$$r^* = \frac{-2\gamma}{\Delta G_v} \quad \text{(SI6)}$$

Where γ is the surface area of the nucleating atom, the ΔG_v is the term of the free energy of the bulk crystal and r^* is the critical radian of nucleation as shown in equation (6).

ΔG°in nucleation mechanism has the direct proportionality with radian of nucleation (r) e.g. the nucleation radian was extended as the ΔG° increases. Another parameter is r^* which is strongly correlated to the size of atomic nucleation to the bulk crystal. Generally, the r has direct change with $\Delta G_{surface}$ and the maximum of $\Delta G_{surface}$ is r^* point which this point showed the highest of $\Delta G_{surface}$ and on the other hand the ΔG_{volume} has indirect change with r extended consequently, ΔG° in this mechanism was combined with $\Delta G_{surface}$ and ΔG_{volume} which the saturated point of both parameters is r* point (r_{critic} of Figure 7 (c) from Choopun *et al*, 2010 [1]) that meaning at r* is the stable state of nucleation atom (highest of $\Delta G_{surface}$ and lowest of ΔG_{volume}) and ready to grow for bulk crystal.

$$\Delta G^{\circ} = \Delta G_{surface} + \Delta G_{volume} \quad \text{(SI7)}$$

Therefore, this growth mechanism favored r^* in determining the probability of the bulk crystal grown. When $r > r^*$ the nucleation atom can perform to bulk crystal, however, if $r < r^*$ the nucleation of bulk crystal is considered thermodynamically less preferred. [1-6].

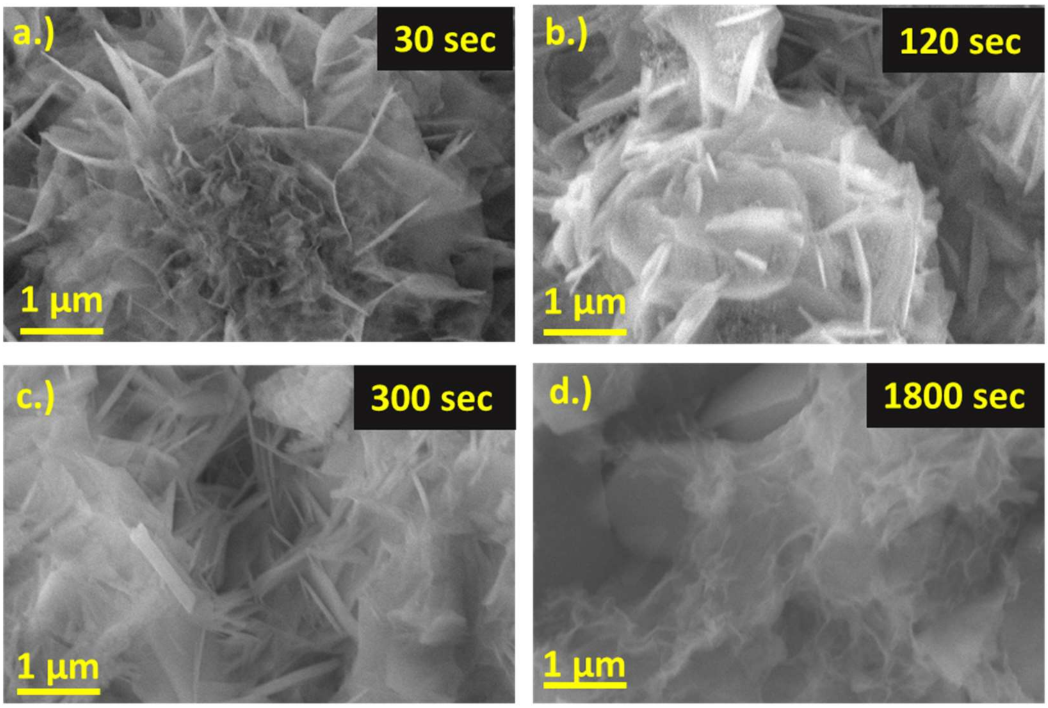

Figure.S2 SEM image of grown h-ZnO flakes on the electrode at a.)30 seconds, b.) 120 seconds, c.) 300 seconds and d.) 1800 seconds into the growth.

In conclusion, the two-step of h-ZnO flakes growth were determined with reaction rate via electron transfer of electrode surface and electrolyte in primary growth process which the low reaction rate as 0.1M of KCl was the reasonable condition to nucleation ZnO crystal after that using secondary growth was the coalescence effect to fusion nucleation grains of ZnO in primary growth for the improved and relatively larger h-ZnO flakes' size in this work.

SII: ZnO electrical measurements

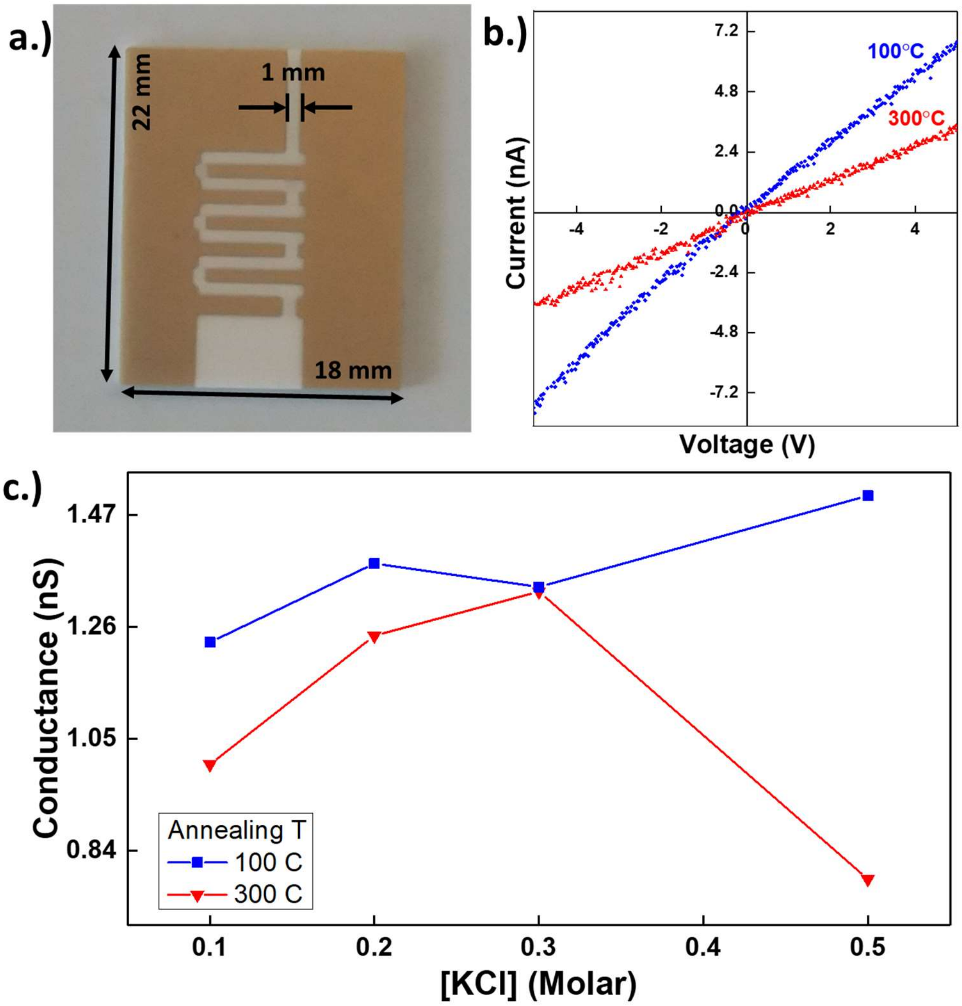

Figure S3. Illustration of a.) the device fabricated for deposited ZnO flakes, b.) I-V curve of the ZnO nanoflakes device with [KCl] = 0.5 M and c.) the calculated conductance of ZnO device vs [KCl] used in the synthesis.

To further demonstrate the capability of the grown material for applications for our proposed growth method, ZnO electrical measurements were conducted. The alumina substrate was lithographed with Au deposition as demonstrated in Figure S3.a.) prior to ZnO deposition via sol-gel process on the prepared substrate. A series of I-V test was conducted on samples annealed at 100 and 300 °C while prepared from various KCl concentration for comparison. The example of our I-V result is shown in Figure S3.b.) where the device demonstrate linear contact with Au. This demonstrates the effectiveness of ZnO prepared via this method as electron collection layer for temperature sensitive Perovskites [7]. From extracted conductance in Figure S3.c.), it is very notable that devices made of flakes annealed at 100 °C has higher conductance compared to the 300 °C ones, this is likely due to the conductance from Cl^- ions remnant which are more available at lower annealing temperature. Although 300 °C annealing should improve crystallinity of the h-ZnO flakes [8-9], with less dopant available in the band gap potentially lowers the conductance given the improved crystallinity. This becomes evident in 0.5M case where Cl^- ions become more dominant prior to

growth but are later eliminated when annealed at higher temperature potentially resulting in more defective channel when used as source and drain contacts.

References for Supplementary Materials

1. Choopun, S.; Hongsith, N.; Wongrat, E., Metal-Oxide Nanowires by Thermal Oxidation Reaction Technique. **2010**.
2. Wu, Z.; Yang, S.; Wu, W., Shape control of inorganic nanoparticles from solution. *Nanoscale* **2016,** *8* (3), 1237-59.
3. Vega-Poot, A. G.; Rodriguez-Gattorno, G.; Soberanis-Dominguez, O. E.; Patino-Diaz, R. T.; Espinosa-Pesqueira, M.; Oskam, G., The nucleation kinetics of ZnO nanoparticles from ZnCl2 in ethanol solutions. *Nanoscale* **2010,** *2* (12), 2710-7.
4. Wang, Z. L., Zinc oxide nanostructures: growth, properties and applications. *Journal of Physics: Condensed Matter* **2004,** *16* (25), R829-R858.
5. Thanh, N. T.; Maclean, N.; Mahiddine, S., Mechanisms of nucleation and growth of nanoparticles in solution. *Chem Rev* **2014,** *114* (15), 7610-30.
6. Lizandara-Pueyo, C.; van den Berg, M. W. E.; De Toni, A.; Goes, T.; Polarz, S., Nucleation and Growth of ZnO in Organic Solvents - an in Situ Study. *Journal of the American Chemical Society* **2008,** *130* (49), 16601-16610.
7. Kim, J.; Kim, G.; Kyun Kim, T.; Kwon, S.; Back, H.; Lee, J.; Ho Lee, S.; Kang, H.; Lee, K., Efficient planar-heterojunction perovskite solar cells achieved via interfacial modification of a sol–gel ZnO electron collection layer. *Journal of Materials Chemistry A* **2014,** *2* (41), 17291-17296.
8. Gao, X.-D.; Li, X.-M.; Zhang, S.; Yu, W.-D.; Qiu, J.-J., ZnO submicron structures of controlled morphology synthesized in zinc-hexamethylenetetramine-ethylenediamine aqueous system. *Journal of Materials Research* **2011,** *22* (07), 1815-1823.
9. Park, S.; An, S.; Ko, H.; Jin, C.; Lee, C., Synthesis of Nanograined ZnO Nanowires and Their Enhanced Gas Sensing Properties. *ACS Appl. Mater. Interfaces* **2012,** *4* (7), 3650-3656.
